# Optimization in the utility maximization framework for conservation planning: a comparison of solution procedures in a study of multifunctional agriculture

**DOI:** 10.7717/peerj.690

**Published:** 2014-12-11

**Authors:** Jason Kreitler, David M. Stoms, Frank W. Davis

**Affiliations:** 1 Western Geographic Science Center, U.S. Geological Survey, USA; 2 Bren School of Environmental Science and Management, University of California Santa Barbara, USA

**Keywords:** Spatial conservation prioritization, Multifunctional agriculture, Conservation planning, Utility maximization, Central Valley, California, Farmland conservation

## Abstract

Quantitative methods of spatial conservation prioritization have traditionally been applied to issues in conservation biology and reserve design, though their use in other types of natural resource management is growing. The utility maximization problem is one form of a covering problem where multiple criteria can represent the expected social benefits of conservation action. This approach allows flexibility with a problem formulation that is more general than typical reserve design problems, though the solution methods are very similar. However, few studies have addressed optimization in utility maximization problems for conservation planning, and the effect of solution procedure is largely unquantified. Therefore, this study mapped five criteria describing elements of multifunctional agriculture to determine a hypothetical conservation resource allocation plan for agricultural land conservation in the Central Valley of CA, USA. We compared solution procedures within the utility maximization framework to determine the difference between an open source integer programming approach and a greedy heuristic, and find gains from optimization of up to 12%. We also model land availability for conservation action as a stochastic process and determine the decline in total utility compared to the globally optimal set using both solution algorithms. Our results are comparable to other studies illustrating the benefits of optimization for different conservation planning problems, and highlight the importance of maximizing the effectiveness of limited funding for conservation and natural resource management.

## Introduction

For conservation to achieve success in a dynamic and changing world, many issues must be addressed. Threats, costs, site availability, and type of conservation action, in addition to biodiversity targets or other conservation benefits, are important factors to consider when allocating limited conservation funds (Pressey et al., 2007; Wilson, Carwardine & Possingham, 2009; Bryan, 2010). Systematic conservation planning (Margules & Pressey, 2000) has traditionally emphasized designing reserves for biodiversity conservation (Moilanen, Wilson & Possingham, 2009). However, tools from spatial conservation prioritization, the prioritization of conservation actions through quantitative means (Moilanen, Wilson & Possingham, 2009), have been applied to prioritize other resources such as ecosystem services (Chan et al., 2006; Chan & Daily, 2008; Bryan, 2010; Bryan et al., 2010), the future ranges of biodiversity (Hannah et al., 2005; Phillips et al., 2008), and multifunctional agricultural lands (Machado et al., 2006; Stoms, Kreitler & Davis, 2011), and for other conservation actions, such as restoration (Crossman & Bryan, 2006; Thomson et al., 2009; McBride et al., 2010) or invasive species control (Wainger et al., 2010).

The problem formulation of many cases of spatial conservation prioritization can generally be defined as trying to find the minimum set solution, the network with the minimum area or cost that meets all of the conservation targets (Possingham & Andelman, 2000; Moilanen, Wilson & Possingham, 2009; Wilson, Cabeza & Klein, 2009), or the maximal coverage solution, the network with the most conservation targets met at a specified budget (Church, 1974; Church, Stoms & Davis, 1996; ReVelle, Williams & Boland, 2002). Utility maximization is one case of the maximal coverage problem (Davis, Costello & Stoms, 2006; Moilanen, Wilson & Possingham, 2009).

Utility maximization problems are similar to maximal coverage problems in conservation planning in that the goal is to maximize the benefit of conservation actions subject to a resource constraint (Moilanen, Wilson & Possingham, 2009). The major difference between the two is in the calculation of site value. In the utility maximization approach, a site’s marginal value is calculated based on the representation level of the resource, and a target amount of the resource, and a benefit or utility function (Davis, Costello & Stoms, 2006). Whereas the maximal covering formulation uses a step function that values all selected sites within the set equally, and non-selected sites have no value. A major advantage of the former is that it allows non-threatened areas outside of the selected set to have value and contribute towards conservation goals. A prime example of a utility maximization problem can be seen in Davis, Costello & Stoms (2006), where conservation funds are allocated to maximize the averted loss of utility, which is a composite of three conservation criteria: hotspots of rare, threatened, and endangered species; under-represented wildlife habitat types; and sites for expanding existing small reserves, all in the Sierra Nevada region of California. They highlight a priority acquisition schedule for conservation and discuss how the framework incorporates key elements of systematic conservation planning (Margules & Pressey, 2000), including concepts of complementarity, efficiency, irreplaceability, and retention (Davis, Costello & Stoms, 2006).

Several recent studies have implemented utility maximization or maximal covering methods for conservation resource allocation problems for purposes other than reserve design (Table 1). This set is not meant to be an exhaustive review, but a representative example of an increasingly common approach in conservation planning. Three of these studies used exact optimization methods like integer programming (IP) or stochastic dynamic programming (SDP), and the rest use a heuristic algorithm or search technique. The use of optimization in conservation planning specifically for reserve selection has shown improvements over simple heuristics (Rodrigues & Gaston, 2002; Williams, ReVelle & Levin, 2004; Haight, Snyder & Revelle, 2005; Moilanen, 2008) with gains from optimization ranging between 5.6%–50% (Pressey, Possingham & Margules, 1996), 4.4%–26% (Csuti et al., 1997), 5%–20% (Onal, 2004), 0%–20% (Fischer & Church, 2005), and 2%–70% (Vanderkam, Wiersma & King, 2007). Similar improvements are likely possible for other types of natural resource management problems that employ spatial conservation prioritization, although the majority of studies based on heuristic methods have not compared solutions to true optimal solutions. Furthermore, an integer programming solution method has not been included within the utility-maximization framework of Davis et al. (2003); Davis, Costello & Stoms (2006), and the difference in results between optimal and heuristic targeting algorithms has not been determined. Alternatively, several studies have illustrated that optimally designed conservation plans may fall short or be unnecessary due to uncertainty in available funding, conservation opportunity, and natural resource degradation in unprotected sites (Costello & Polasky, 2004; Meir, Andelman & Possingham, 2004; Pressey et al., 2007).

**Table 1:** Comparison of 16 utility maximization or maximal covering problems in conservation. Characteristics of utility maximization or maximal covering methods for conservation resource allocation problems.

Conservation objective	Problem formulation	Solution procedure	Benefit measure	Utility function	Threat	Conservation action cost	Reference	Year
Ecosystem services	Max benefits subject to budget	Heuristic	MC weighted sum	CM	No	Opportunity cost	Hajkowicz et al., 2005	2005
Restoration planning	Min restored sites subject to targets	IP	Vegetation targets	CM	No	Area	Crossman & Bryan, 2006	2006
Biodiversity	Max utility subject to budget	Heuristic	MC weighted sum	CUF	Development	Acquisition	Davis, Costello & Stoms, 2006	2006
Multifunctional ag	Max utility subject to budget	Heuristic	MC weighted sum	CUF	Development	acquisition	Machado et al., 2006	2006
Open space & habitat	Max benefits subject to budget	SDP & Heuristic	MC weighted sum	SF	Development	Easement	Newburn, Berck & Merenlender, 2006	2006
Biodiversity	Max sp. At end horizon	SDP & Heuristic	Marginal species gain	SAR	Species loss	Acquisition	Wilson et al., 2006	2006
Biodiversity	Max species persistence	Heuristic	Marginal species gain	SAR	Species loss	Multiple actions	Murdoch et al., 2007	2007
Biodiversity	Max species persistence	Heuristic	Marginal species gain	SAR	Species loss	Multiple actions	Wilson et al., 2007	2007
Biodiversity	Max species subject to budget	Heuristic	Marginal species gain	SAR	No	Acquisition	Underwood et al., 2008	2008
Ecosystem services	Max benefits subject to budget	Heuristic	MC weighted sum	SF	Deforestation	Opportunity & mgmt	Wuenscher, Engel & Wunder, 2008	2008
Ecosystem services	Max cost effectiveness subject to area	Heuristic	MC weighted sum	CM	No	Opportunity cost	Crossman & Bryan, 2009	2009
Biodiversity	Max species subject to budget	Heuristic	Marginal species gain	SAR	No	Acquisition	Underwood et al., 2009	2009
Ecosystem service	Max ES value subject to water amount	Heuristic	Value of ES	CM	No	Value of ES	Crossman et al., 2010	2010
Multifunctional ag	Max utility subject to budget	Heuristic	MC weighted sum	CUF	Development	Acquisition	Stoms, Kreitler & Davis, 2011	2011
Ecosystem services	Max benefits subject to budget	GA	MC weighted sum	CM	No	Managmenet cost	Wainger et al., 2010	2010
Restoration planning	Max restoration benefits	Heuristic	Vegetation targets	CUF	No	No	Thomson et al., 2009	2009

**Notes.**

CM Individual criteria models CUF Convex utility function SAR Species area relationship SF Step function IP Integer programming SDP Stochastic dynamic programming GA Genetic algorithm

In previous research using the utility-maximization approach in an agricultural context, Stoms, Kreitler & Davis (2011) evaluated the efficiency of solutions based on the types of data used and how a cost-effectiveness score was calculated to maximize utility in preserving multifunctional farmland. For our purposes we define multifunctional agriculture in a general sense as a land use jointly producing multiple commodity and non-commodity outputs, where some of the non-commodity outputs may be public goods with non-existent markets, and thus be undersupplied without intervention (Renting et al., 2009). We quantify multifunctionality in our example through spatial models of individual criteria representing functions of the agricultural landscape that produce public or private goods. These criteria are agricultural viability, priority conservation areas, conservation buffers, sphere of influence, and flood liability (described in sections “Combined benefit criteria” –“Sphere of influence” below).

In this paper, we compared an optimal integer programming (IP) model to the best performing solution procedure from Stoms, Kreitler & Davis (2011), in the allocation of farmland conservation funds in the Central Valley of California. We quantified the difference in benefits accrued (accumulated utility) between solutions to compare the gains produced by a simple algorithm (greedy heuristic) with the optimal solution in the utility maximization framework of Davis et al. (2003) and Davis, Costello & Stoms (2006). In addition, we quantified the difference in benefits when parcel availability was a stochastic process more reflective of real estate markets and actual land-use change through time, and compared results to the global solution where all parcels were available.

## Methods

### Case study of Sacramento & San Joaquin counties

**Figure 1:**
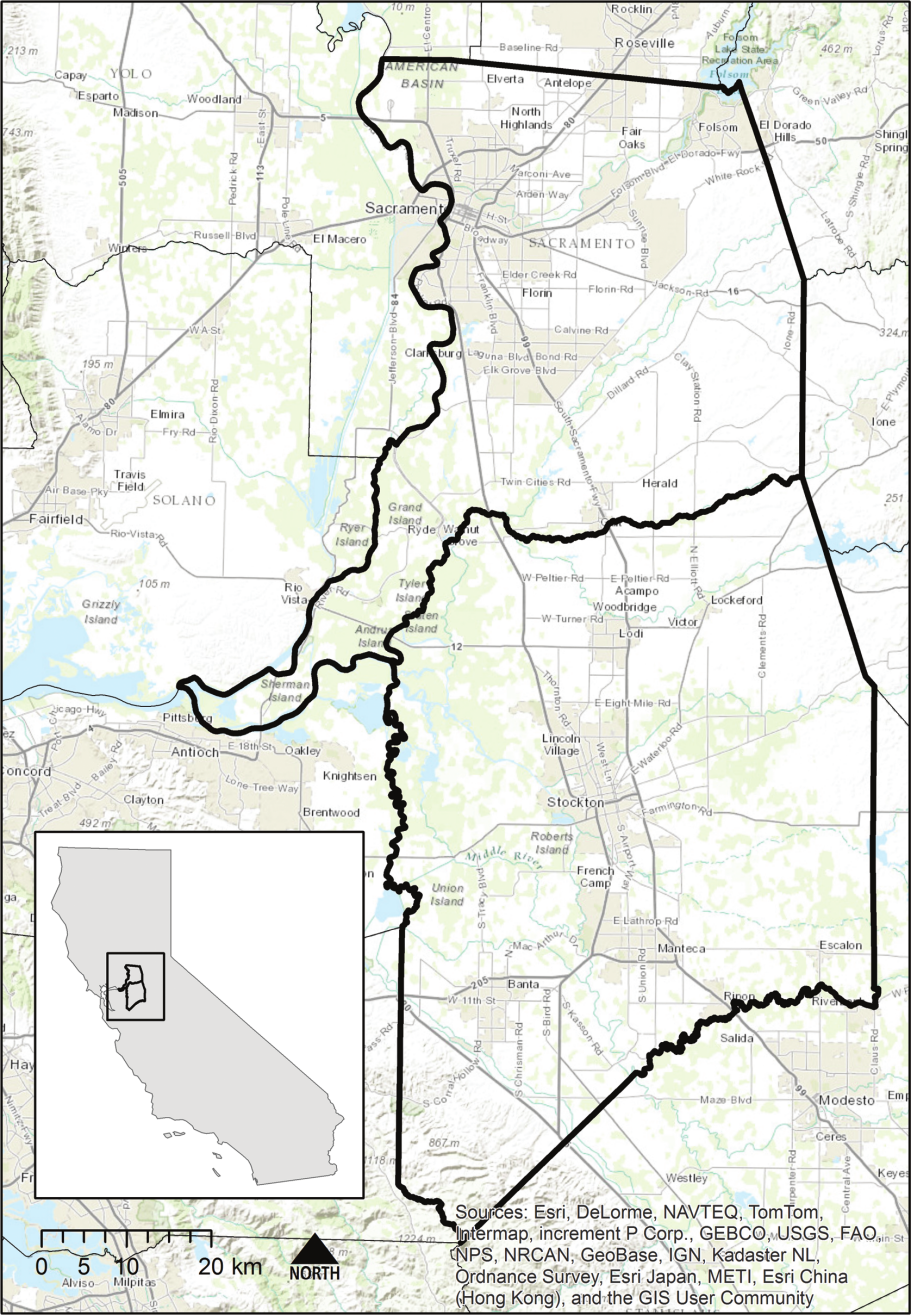
Location of the study area. Study area of Sacramento and San Joaquin Counties (bold outlines), within California (inset), USA.

Our study area is delineated by the political boundaries of Sacramento and San Joaquin Counties (Fig. 1). This area contains two major cities, Sacramento and Stockton, and many other growing San Francisco Bay Area commuter towns and agricultural centers. The 6,26,730 ha area is comprised of 49.5% agricultural lands, 23% natural land cover (forests, shrublands and grasslands), 21.5% developed area, and 5% wetland and open water. Between 1992 and 2008, 29,012 ha of agricultural lands were lost to urban development, primarily at the urban fringe. In Sacramento County, equal amounts of grassland and farmland were converted to urban development, while in San Joaquin County more than 75% of new urban land had been farmland. In addition to the economic and cultural impacts, loss of farmland in this region has implications for natural resource protection, as many of these lands would be considered multifunctional. Biodiversity is negatively affected by urban growth, and a large portion of the region with the potential for urban development occurs in low-lying areas with the possibility of catastrophic floods. Due to recent court rulings (Arreola v. County of Monterey; Paterno v. State of California), the State will ultimately be liable for flood damages (Fridirici, 2008; Duane, 2010) and potential economic damages to urban development are much greater than to agricultural land (Fridirici, 2008; Duane, 2010). Recent farmland losses in the region have prompted governmental and non-governmental action to preserve farmland from development. In San Joaquin County a farmland mitigation fee was established in an attempt to mitigate farmland losses. The Central Valley Farmland Trust, a non-profit land trust, was also established to facilitate the preservation of valuable agricultural land.

### Utility maximization in multifunctional agriculture conservation

We used the utility maximization approach (Davis, Costello & Stoms, 2006; Machado et al., 2006) to allocate farmland conservation funds. The goal of this approach is to allocate farmland conservation funds to maximize the averted loss of multifunctional farmlands within the planning region through parcel acquisition. Without consideration of expected loss, funds may be ineffectively used to conserve land not likely to be converted (Newburn et al., 2005). We used the California Urban and Biodiversity Analysis model (CURBA, Landis & Reilly, 2002). to project urban growth in the region. Our approach also included the cost of conservation acquisition (section “Cost modeling”) to ensure resources were allocated cost-effectively. Each parcel received a utility score based on five criteria representing characteristics of multifunctional agriculture (sections “Combined benefit criteria”–“Sphere of influence”), and a utility function (section “Combined benefit criteria”). We then used two prioritization algorithms to select and compare sets of prioritized parcels for a given budget. Cost-effectiveness is calculated as a site’s utility score divided by the modeled acquisition cost to secure that utility from expected future loss.

#### Combined benefit criteria

We converted the five agricultural conservation criteria (agricultural viability, priority conservation areas, conservation buffers, flood liability, and sphere of influence, described below and in Machado et al. (2006) and Stoms, Kreitler & Davis (2011) for more detail), to marginal utility values before combining them through a weighted-linear combination (Malczewski, 1999). In this example the criteria are equally weighted, but user-defined weights could be applied to reflect varying social preferences for the function of agriculture. This takes the form:

(1) \begin{eqnarray*} {B}_{x}=\sum _{j}{w}_{j}{f}_{j}[{R}_{j}(x)] \end{eqnarray*}

where the combined utility (*B_x_*) is the sum of the products of each criterion-specific weight (*w_j_*) and *f_j_* [*R_j_*(*x*)], which is the marginal value of *x* according to utility function *f_j_* and representation level *R_j_*. *f_j_*_ _ can take many functional forms, and this example we used a concave utility function as in previous studies (Davis, Costello & Stoms, 2006; Machado et al., 2006; Stoms, Kreitler & Davis, 2011). The representation level (*R_j_*) is the amount of the region specific non-threatened resource measured in criterion _*j*_.

#### Agricultural viability

We used a measure of agricultural viability to represent the potential for continued agricultural production on a given parcel. Agricultural viability was the product of estimated agricultural production capability and a measure of local condition (Machado et al., 2006). Production capability is a function of soil quality, climate, and water supply. Local condition was based on fragmentation of nearby lands and declines as urban land uses extend into agricultural lands (Bradshaw & Muller, 1998; Machado et al., 2006). To project agricultural viability in the future and model potential loss, we adjusted the production capability such that parcels projected to be developed had no production capability and local condition was updated based on the new urban frontier.

#### Priority conservation areas

We obtained data on priority conservation areas from a biodiversity conservation organization describing agricultural areas of interest for acquisition or conservation management. These zones integrate biodiversity conservation planning activities and serve as a proxy for a biodiversity conservation criterion. Within our study area there were eight separate areas ranging in size from 61 ha to 112,716 ha that were considered high conservation priority. Parcels were scored based on the amount of area falling within a priority conservation area that was also expected to be lost to development. While this criterion is not dynamic from the perspective of ensuring all species within the original conservation planning activities are covered, it is updated when conservation actions occur. Therefore, if a large portion of one priority conservation area is secured, the marginal value of the remaining area within that priority conservation area will be adjusted according to utility function, targets, and existing protected resources.

#### Conservation buffers

The conservation buffer criterion scored parcels based on the area contained within a parcel that fell within a one kilometer buffer around existing conservation lands. This criterion is based on the premise that areas immediately adjacent to reserves increase the effectiveness of the reserve by reducing the ecological contrast and associated edge effects between reserves and non-reserves and by providing additional habitat for wide ranging species, particularly for small reserves in working landscapes (Wiens, 2009).

#### Flood liability

The flood liability criterion prioritizes areas where farmland conservation would act as a flood risk reduction strategy by keeping urban development out of areas expected to flood (Kousky et al., 2013; Kousky & Walls, 2014). It is calculated by intersecting expected development and area within the hundred year floodplain. The CURBA model and an Army Corps of Engineers study (2002) were used to determine a parcel’s area in each zone. Parcels scoring high for this criterion have farmland within floodplains where land use conversion was expected.

### Sphere of influence

In California, local agency formation commissions (LAFCO) are required to delineate a sphere of influence surrounding incorporated areas that encourage orderly growth, preserve agricultural lands, and discourage urban sprawl (Fulton, 1999). The sphere of influence criterion scored parcels that are within a one kilometer buffer of spheres of influence around existing communities. In this manner, agricultural conservation was prioritized as a growth management tool to direct urbanization away from conservation resources (Stoms et al., 2009).

### Cost modeling

Information on the cost of potential conservation actions plays a critical role in efficient conservation planning (Ando et al., 1998; Naidoo & Adamowicz, 2006; Naidoo & Ricketts, 2006; Newburn, Berck & Merenlender, 2006; Polasky, 2008; Stoms, Kreitler & Davis, 2011). In a typical conservation acquisition, resources are preserved through a conservation action, usually in the form of a purchase of property or development rights. With this action the resources are assumed to be protected from future loss. In some areas accurate data on land values are available, commonly through the local property tax assessor. In many areas the data are not accurate or available, so they must be modeled from observed transactions. Many studies rely on county or regionally averaged land values to determine the unit price of land (Polasky, Camm & Garber-Yonts, 2001; Davis, Costello & Stoms, 2006; Carwardine et al., 2008). Yet due to the common positive correlation between land cost and development threat (Ando et al., 1998), county averaged land values will likely underestimate the cost required to secure threatened resources, biasing the results of conservation plans. We therefore modeled the fine scale heterogeneity in potential acquisition costs at the parcel level to capture gradients of land value.

We modeled land value at the parcel level using farmland property transactions acquired from the Dataquick real estate database service (www.dataquick.com). Using the hedonic pricing function of Rosen (1974) we estimated a predicted price per hectare within the two county study area. The potential value of urbanization of the parcel was capitalized in the market price. Variables from our model that explain per hectare variation in land value included parcel size, distance to urban areas, allowed uses, and presence within the one hundred year floodplain (*p* < 0.0045, *r*^2^ = 0.64).

### Stochastic simulation

Scheduling and land use change can impact the success of conservation actions (Armsworth et al., 2006; Wilson et al., 2006; Wilson et al., 2011), therefore, we simulated parcel availability as a stochastic process to determine the difference in accumulated utility when available sites were less than the full set (*n* = 31,020). A parcel availability matrix of 1,000 parcels by 4,000 simulations (40 time steps per 100 budget scenarios) was created with modeled probability of availability decreasing with increasing distance from urban centers, reflective of observed land use transitions in the region (Stewart & Duane, 2009; Duane, 2010). Selected (conserved) parcels were unavailable in subsequent time steps. Both solution procedures in the stochastic scenario drew from the same matrix of parcel availability at each step, therefore direct pairwise comparison of solution procedures is possible.

### Solution procedures and comparison

Using our dataset of multifunctional farmland utility we compared four scenarios. The IP and greedy heuristic solutions could select any parcel in the dataset, in any time period if the parcel had not already been prioritized for conservation action. We also performed both methods with a constraint of 1,000 available parcels in the land market at each time period (stochastic IP and stochastic greedy), to examine a situation more representative of what a conservation organization would face. All procedures follow the form:

1. Convert criteria scores to combined marginal values at each site (Eq. (1)).
2. Target sites to maximize the sum of *B*(*x*) subject to an annual budget (both IP and greedy).
3. Recalculate representation levels for each criterion (*R_j_*).
4. Recalculate marginal values at all unselected sites.
5. Continue steps 2–4 for a specified time period or total budget amount.
6. Calculate total utility of the selected set for comparison.

Both solution procedures were written in the R statistical modeling environment (R Development Core Team, 2010). More detail on incorporating the IP solver into the utility maximization approach is included as pseudocode in Appendix S1. The IP formulation used R package lpSolve with the lpSolve program (Berkelaar, 2008), a free open source linear and mixed integer problem solver that uses the Simplex algorithm (Danzig, 1998). This solver was used, as opposed to commercial software, to demonstrate a completely open source software solution. The greedy heuristic sorted the cost effectiveness score in descending order and selected sites until the budget constraint was met each time period. We modeled cost effectiveness as a site’s marginal value divided by its modeled acquisition cost.

To compare the similarity of selected sets we used the Jaccard similarity index (Jaccard, 1901), an index commonly used in ecology to describe community similarity between pairs of samples based on the presence or absence of species in each sample. The Jaccard similarity index is calculated as the intersection of the two sets, divided by their union and ranges from 0 to 1.

## Results

### Spatial heterogeneity of multifunctional agricultural lands

**Figure 2:**
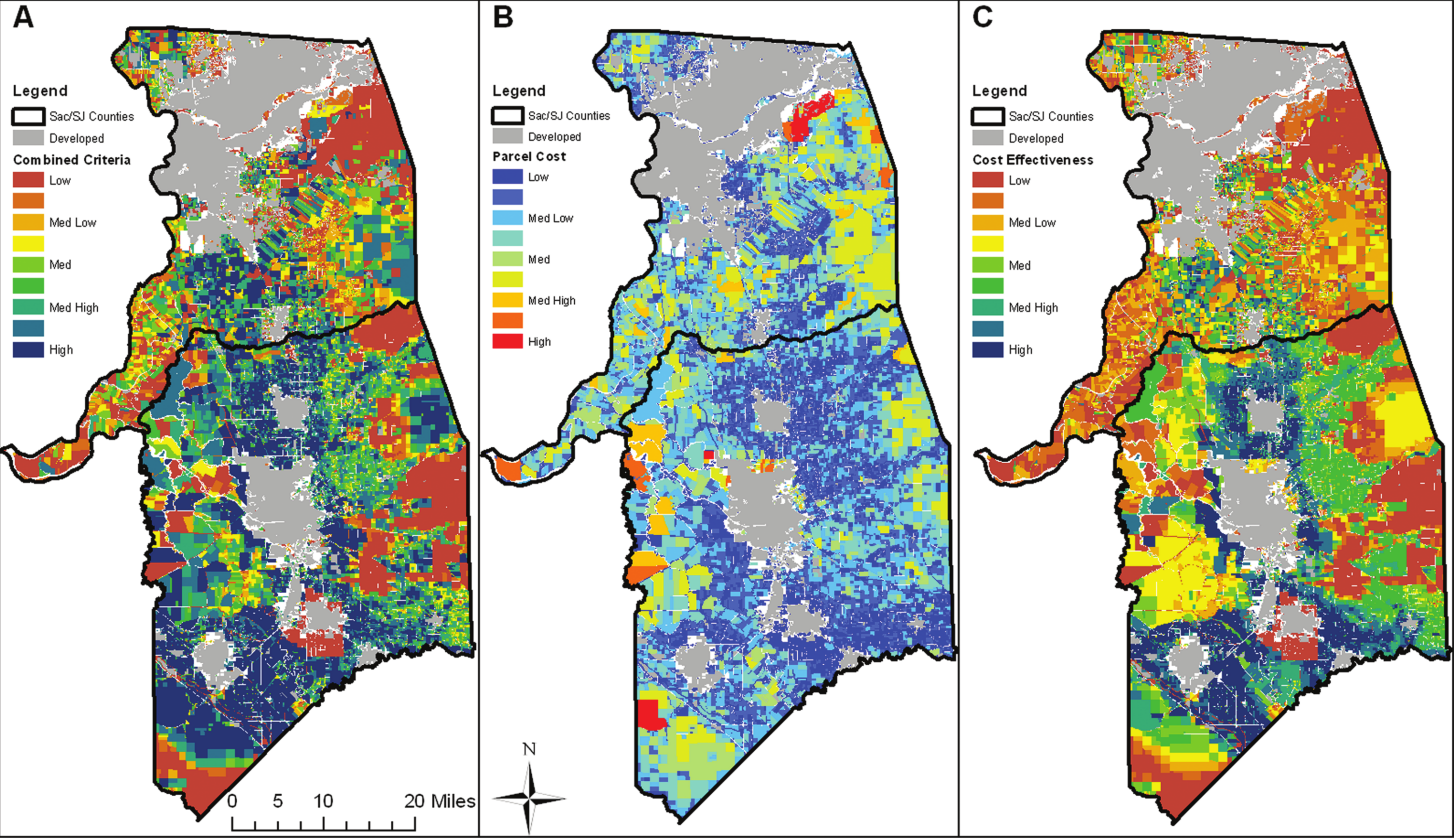
Benefit, cost, and cost-effectiveness data. Heterogeneity of combined criteria benefits (A), modeled parcel acquisition costs (B), and cost-effectiveness of each parcel, calculated as benefits/costs (C).

The combined utility score, modeled parcel acquisition price, and cost-effectiveness results are illustrated in Fig. 2. The equally weighted combined criteria score (Fig. 2A) shows areas of high net benefit for multifunctional agricultural lands around the developed areas in the southern portion of the study area (near the cities of Lodi, Stockton, and Tracy (Fig. 1)). This is likely due to the presence of prime agricultural lands, flood risk, and strategic position between communities and development threat. In Fig. 2B, parcel acquisition prices were largely dependent on parcel size and distance from urban areas. Parcel size generally increases with increasing distance from urban areas, so acquisition prices largely increase as distance from urban increases, even though per acre price is generally highest near the urban edge and declines with increasing distance. Figure 2C shows the cost-effectiveness of each parcel as the ratio of combined utility divided by modeled acquisition costs. Even with the higher per-acre cost factored in, the areas around Lodi, east and west of Stockton, surrounding Tracy, and to the east of Manteca have high cost-effectiveness. The majority of cost-effective parcels are located within San Joaquin County, though parcels are selected in southern Sacramento County as well.

### Comparison of solution procedures

**Figure 3:**
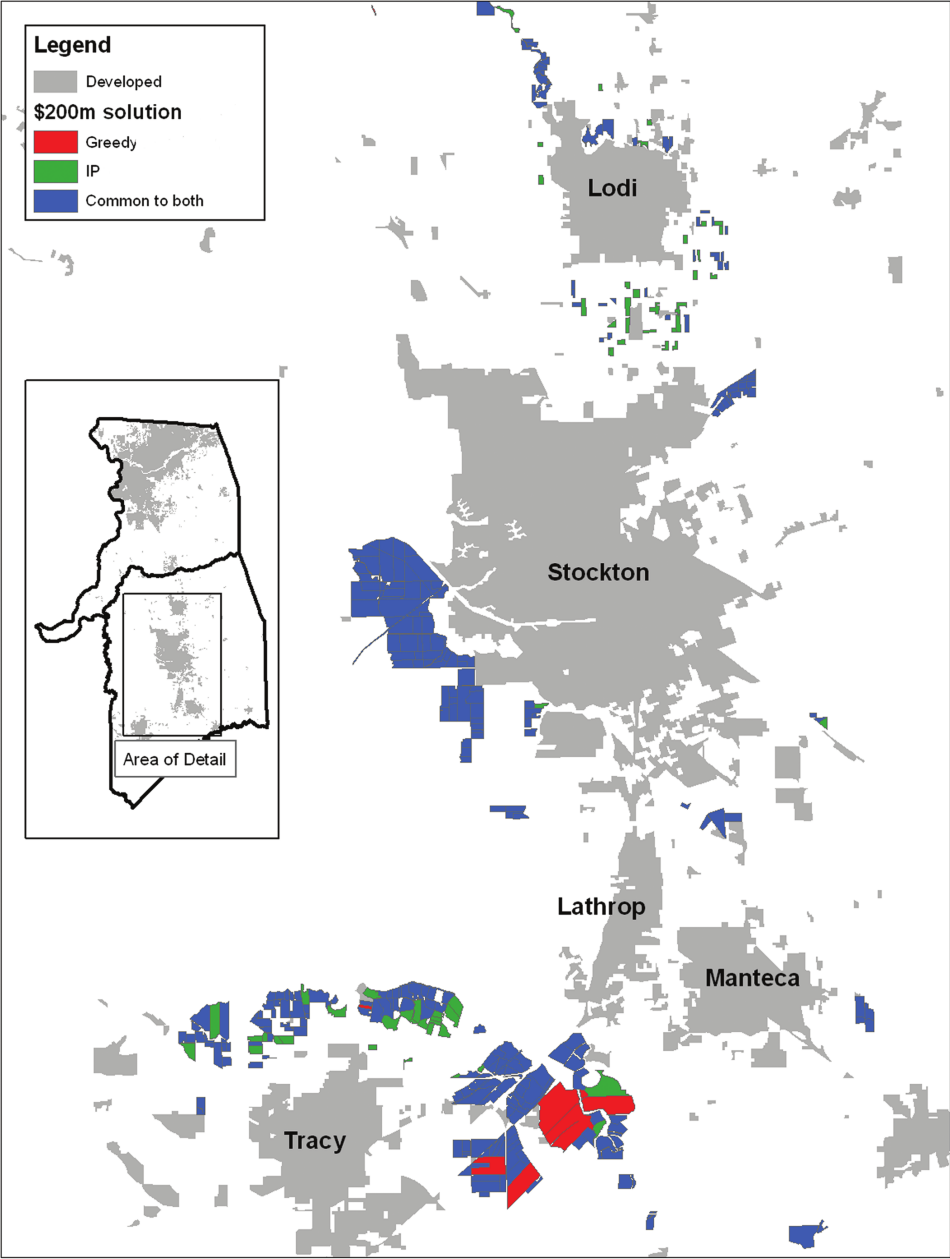
Divergence and agreement between solutions. Comparison of the $200 million ($US) scenario results for a subset of the study area where a large number of parcels are prioritized by both the greedy and IP solution procedures.

The selected sites both in the IP and greedy solution at the $200 million budget are relatively clustered and compact due to the spatial characteristics of the criteria models (Fig. 3). The two methods select relatively similar sets (Jaccard similarity = 0.89). However, a major difference between the IP and greedy procedures is that the sites unique to the IP solution are more numerous and smaller, whereas fewer, larger sites are selected solely by the greedy solution method (Fig. 3).

**Figure 4:**
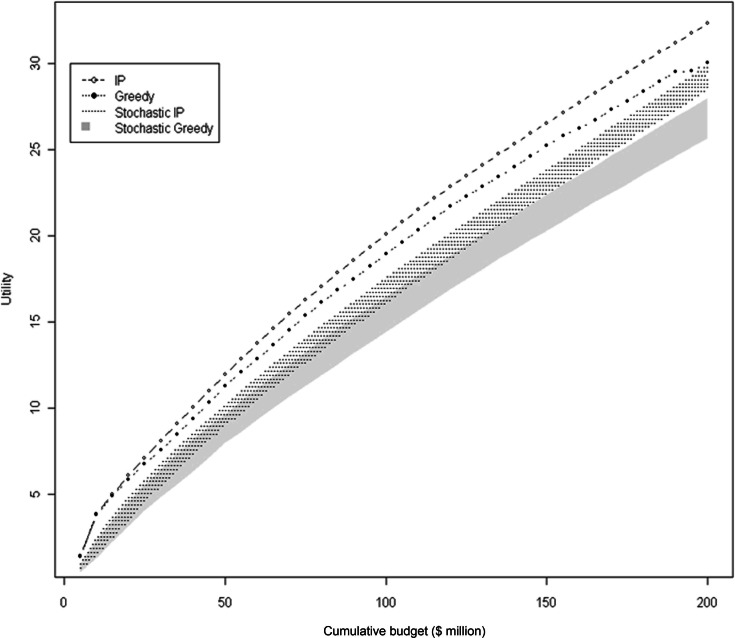
Comparison of solution procedures by total utility and cumulative cost. Accumulated utility by solution procedure for level of cumulative expenditure. In the stochastic simulations, the shaded areas represent the ranges of values for each solution procedure.

A comparison of the total accumulated utility by solution procedure is illustrated in Fig. 4. The IP and greedy solutions begin to diverge once $20 million is spent, with the difference between IP and greedy gradually increasing until the total budget is spent at $200 million. By the end of the budget the IP solution has accumulated 7% more utility than the greedy. Accumulated utility increases sharply from the beginning of the selection in both procedures, and then tapers as sites with lower marginal utilities are selected.

When the potential sites are limited to those hypothetically available at each time interval (1,000 of the 31,020), the total accumulated utility ranges from 88% to 70% of the optimal IP solution for the stochastic IP and greedy solutions, respectively. The distributions of utility for the stochastic solutions overlap at the start but are significantly different at all intervals, (paired *t*-test, *t* = −3.077, *p* < 0.002), and then completely separate by the end of the scenario. The mean difference in utility between solution procedures by the end of the budget is 8% (ranging between 5 and 12%), with a mean pairwise Jaccard similarity of 0.86 (ranging between 0.82 and 0.91).

## Discussion

This study mapped five criteria describing elements of multifunctional agriculture to determine a hypothetical conservation resource allocation plan for agricultural land conservation. We compared solution procedures to determine the difference between an optimal integer programming approach and a greedy heuristic in a utility maximization problem. Stoms, Kreitler & Davis (2011) showed the importance of including benefit-loss-cost data in this study area; in this effort we demonstrate the improvement found through optimization methods, and determine the decline in total utility when site availability is a stochastic process.

Our results show that the optimal solution and the greedy solutions were similar, but in each case, the IP formulation outperformed the greedy heuristic by up to 12%. We have described the solution of a knapsack or packing problem (Martello, Pisinger & Toth, 2000; Caccetta & Kulanoot, 2001), in a utility maximization approach (Davis, Costello & Stoms, 2006; Wilson et al., 2006; Moilanen, Wilson & Possingham, 2009), where the IP formulation found an incrementally more efficient solution given the budget constraint. This can be observed in Fig. 3 by comparing the selected sites unique to each solution method. The IP solution used numerous small, less expensive sites to maximize the utility, whereas the greedy solution was 7% less effective by simply selecting parcels in order of decreasing cost-effectiveness. Another noteworthy result is the proximity of the upper limit of the stochastic IP result to the greedy result at the $200 m level (Fig. 4). Even with reduced site availability, the IP solution approaches the full greedy solution that had access to all sites in the study area. This result may be partially due to the interchangeability of sites as well as the IP solver finding better solutions.

The methods described here have potential implications for many natural resource management applications. The papers reviewed in Table 1 all use a similar mathematical formulation in which an objective function is maximized subject to a budget constraint. Most do not employ optimization methods and could likely be improved by adopting optimal solution procedures as illustrated here, and reviewed elsewhere (Rodrigues & Gaston, 2002; Williams, ReVelle & Levin, 2004; Haight, Snyder & Revelle, 2005). Furthermore, future studies could utilize the accessibility of lpSolve in the flexible R modeling environment without having to rely on proprietary commercial software.

This study is unique in its combination of integer programming and a benefit or utility function approach. We are not aware of the use of IP or other search methods within utility maximization planning for multiple criteria. Wilson et al. (2006) used optimization methods with a benefit function, but for the singular objective of maximizing species persistence by scheduling ecoregional conservation actions through space and time. The use of multiple criteria allows a broader suite of potential applications in restoration planning or ecosystem service conservation planning, for example. Instead of a utility function, process based models (Ferrier & Drielsma, 2010) or ecosystem service production functions (Nelson et al., 2008; Nelson et al., 2009) could be included to convert landscape patterns into restoration benefits or ecosystem service values for conservation resource allocation schedules that are reflective of social preferences.

Perhaps the largest drawback of using IP is the linear constraint on problem formulation. This “first stage suboptimality” (Moilanen, 2008) occurs when a complicated problem is simplified to fit into a linear formulation for which there is a tractable globally optimal solution. In our methods we take this approach by simplifying our criteria models. For example, our models calculate initial criteria values according to subsections “Agricultural viability”–“Flood liability” and incorporate distance and contiguity measures. These criteria scores are converted to marginal utility values per Eq. (1) and dynamically updated after each round of conservation actions, but the spatial operations are not, which leads to a more tractable problem for optimization. In this paper we have measured Moilanen’s (2008) “second stage suboptimality”, or the difference between the exact IP and heuristic solutions. However, for the reasons described above there may be larger true differences between linearly formulated exact IP solutions and heuristic solutions found using more complicated and realistic nonlinear model formulations, even if a less accurate heuristic algorithm is used to solve the problem.

By contrast, when the results of this study are compared to Stoms, Kreitler & Davis (2011), which used the same data and similar methods to conduct a sensitivity analysis, the largest benefit to maximizing utility is found through using the cost-effectiveness of conservation actions, rather than by including threat estimates or using optimal solution methods. Similar studies have shown the degree of suboptimality from the absence of threat data or from the solution algorithm is of comparable magnitudes to those found here (Moilanen & Cabeza, 2007; Moilanen, 2008).

All of the examples from Table 1 that use the utility maximization approach of Davis, Costello & Stoms (2006) have occurred in regions of California where variation in land value may be a principle factor influencing conservation opportunity. This is important to note when considering the findings, and how generalizable they may be. One of the unique features of this study is the fine scale at which the costs and benefits were calculated, and the spatial correlation of threats from urban development and land value. In regions with less heterogeneity in land value, results would likely be driven more by the benefit surfaces than in this example. Similarly, differences between solution methods would likely diminish as the planning unit scale increases and the decision space decreases to a fewer number of planning units to choose from. Further sensitivity analysis on the selection of land use change scenarios would be a last step in determining the robustness of results to the data inputs required by this planning framework.

The merits and shortfalls of pursuing optimality in conservation planning have received considerable attention, usually with reference to minimum set or maximal covering reserve selection problems (ReVelle, Williams & Boland, 2002; Moilanen, 2008). The framework of utility maximization planning, however, is different due to site values that are typically a function of increasing representation with nominal target values, and the ability to incorporate multiple criteria measured in different units (Davis, Costello & Stoms, 2006; Moilanen, Wilson & Possingham, 2009). The utility or benefit function actually makes utility maximization problems less suitable for IP, though we have described a stepwise procedure to accommodate the numerical requirements of IP and the problem formulation of multicriteria utility maximization planning. The existing conservation planning program Zonation (Moilanen, 2007), with its predefined benefit functions, could be particularly useful for utility maximization problems. Further similarities between the utility maximization approach and Zonation include the ability to incorporate several important data inputs including benefit, threat, and cost data; an iterative heuristic similar to our greedy heuristic; and a built in routine to dynamically update all sites’ marginal values after conservation action. Continued research into the application of these methods and different software solutions is warranted due to the increasing opportunity for use in applied conservation and natural resource management problems in ecosystem services, restoration planning, or multifunctional agriculture conservation prioritization, in addition to biodiversity conservation.

## Supplemental Information

10.7717/peerj.690/supp-1 Appendix S1 Pseudocode using lp_solve
