# Supplementary material for: Optimization in the utility maximization framework for conservation planning: a comparison of solution procedures in a study of multifunctional agriculture
Source: PeerJ. 2014 Dec 11;2:e690. doi: 10.7717/peerj.690 (PMC4266854; doi:10.7717/peerj.690)
Supplement: Appendix S1 [file peerj-02-690-s001.docx]

Appendix 1: Pseudocode using lp_solve

Our utility maximization problem is solved using the integer programming solver lp_solve according to the following pseudocode and data inputs. A thorough user’s guide and user community for lp_solve can be found at http://lpsolve.sourceforge.net/.

Data:

Criteria models measure the benefit contained within each planning unit (sections 2.2.2 – 2.2.5). For each criteria and reference region, present and goal resource amounts are known. These amounts are used to determine the marginal value of each planning unity with the utility curve according to equations 3-5 in Davis et al. (2003). Our utility function is a diminishing quadratic function, with a linear first derivative. Marginal values are then combined according to equations 3. These planning unit resource amounts, marginal values, and their costs make up the data matrix used in the prioritization routine.

Variables:

i = loop control variable

j = number of iterations or budget intervals

budget = new amount per [i]

budget [i] = budget remainder from [i-1]+ budget[i]

R Pseudocode:

FOR (i in 1:j) {

DO <marginal value function on resource amount>

DO <combine marginal values>

DO { # start lp_solve solver

f.obj= c(combined criteria vector)

f.con= matrix(c(planning unit cost vector),nrow=1,byrow=TRUE)

f.dir= c("<=")

f.rhs= budget[i]

ip= lp("max", f.obj, f.con, f.dir, f.rhs, all.bin=TRUE) # “ip$solution” = selected set

} # end lp_solve solution

DO <update present resource amounts>

DO <calculate utility>

DO <mark selections & remove from selectable units>

} # end iteration [i]

Reference cited:

Davis, F.W., D.M. Stoms, C.J. Costello, E.A. Machado, J. Metz, R. Gerrard, S. Andleman, H. Regan, and R. Church. (2003). A framework for setting land conservation priorities using multi-criteria scoring and an optimal fund allocation strategy. Report to the Resources Agency of California. https://www.nceas.ucsb.edu/nceas-web/projects/4040/TerrBiod_framework-report.pdf
